# Characterization of yam virus X isolates from *Dioscorea trifida* in Brazil

**DOI:** 10.1186/s13104-026-07987-0

**Published:** 2026-09-02

**Authors:** Cintia Paula Feitosa Souza Araújo, Dennis Knierim, Saulo Alves Santos de Oliveira, Rosana Blawid, Paolo Margaria

**Affiliations:** 1 Plant Virus Department, Leibniz Institute DSMZ-German Collection of Microorganisms and Cell Cultures GmbH, Inhoffenstrasse 7B, 38124 Brunswick, Germany; 2 Embrapa Mandioca e Fruticultura, Rua Embrapa S/nº, Chapadinha, Bahia 44380-000 Cruz das Almas, Brazil; 3 Department of Agronomy, Federal Rural University of Pernambuco, Rua Dom Manuel de Medeiros S/nº, Dois Irmãos, Pernambuco 52171-900 Recife, Brazil

**Keywords:** Plant virus, Potexvirus, South America, Bioinformatics, Illumina sequencing

## Abstract

**Objective:**

Yam virus X (YVX; *Potexvirus ecsdioscoreae*) is a positive-sense, flexuous RNA virus belonging to the family *Alphaflexiviridae*. It has been first reported from Guadeloupe, a French archipelago located in the Caribbean Sea. In this study, we investigated the virome in yam (*Dioscorea* spp.) plant material collected in the state of Bahia (Brazil) by high-throughput sequencing (HTS) on Illumina platform. The objective of the investigation was to explore the occurrence of YVX in yam from South America, and to study its genetic diversity compared to the only one YVX genome sequence available in the GenBank public database.

**Results:**

An initial investigation by HTS of bulked RNA extracts (n=23, combined into 4 pools) revealed occurrence of YVX only in samples collected in the region of Valença. Subsequent screening by RT-PCR of the individual samples composing the pool uncovered infection with YVX only in *Discorea trifida.* Total RNA extracts from three infected plants were individually sequenced, resulting in the assembly of three complete genome sequences of YVX, showing ~84% nucleotide identity to the reference sequence from Guadeloupe. Our results contribute to expanding the pool of sequences available for YVX, supporting detection purposes and stimulating additional investigations for future studies on YVX diversity and evolution.

**Supplementary Information:**

The online version contains supplementary material available at https://doi.org/10.1186/s13104-026-07987-0.

## Introduction

Yam is a major staple food crop in West Africa, which accounts for the majority of global yam production, and it is also cultivated in several regions across Asia, South America, and Oceania [1]. In Brazil, the main cultivated species of *Dioscorea* include *D. alata*, *D. bulbifera*, the *D. cayennensis-rotundata* complex, and *D. trifida* [2, 3]. Like other *Dioscorea* species, *D. trifida* exhibits genetic diversity among cultivated accessions [4], and local varieties are preserved by farmers in different regions of the country, with selection influenced by regional preferences [5] and local edaphoclimatic conditions.

Due to vegetative propagation of yam through tubers, viruses tend to accumulate over successive cultivation cycles [6, 7]. In Brazil, different viruses have already been reported infecting *Dioscorea* spp., including yam mosaic virus (YMV, *Potyvirus yamtesselati*) [2], yam mild mosaic virus (YMMV, *Potyvirus yamplacidum*) [8], Dioscorea mosaic-associated virus (DMaV, *Sadwavirus dioscoreae*) [9], and members of species belonging to the genus *Badnavirus* [10–12]. Nevertheless, the viral diversity associated with *Dioscorea* spp. in the country remains poorly investigated.

Among the viruses reported infecting yam, yam virus X (YVX) has attracted our attention due to the limited genomic information currently available and its restricted geographic record. YVX is a member of the species *Potexvirus ecsdioscoreae.* The genome of members of the genus is composed of a single linear molecule of positive-sense RNA of 5.9–7.0 kb, with a cap at the 5´ terminus and a polyadenylated tract at the 3′-terminus [13]. The genome has five Open Reading Frames (ORFs), which in direction 5´ to 3´ are coding for: a replication-associated protein (Rep; the viral replicase), three proteins (TGB1, TGB2, TGB3) involved in cell-to-cell movement, and the coat protein (CP) [14].

YVX has been previously reported from Gaudeloupe in *Dioscorea trifida* and in members of the complex *D. cayenensis-rotundata* [15]. Up to date (GenBank access on 15.05.2026), only one complete genome sequence is available in the GenBank public database (accession KJ711908, isolate T551, assigned as NC_025252 reference sequence), together with eight accessions representing partial coding sequences of the viral replicase region. Inspection of the GenBank database, also revealed five accessions from *D. alata* and *Dioscorea opposita* from China assigned to YVX (no. KJ789131, KJ789132, KJ789133, KJ789134, KM009120). However, a sequence comparison in the context of this study, considering the criteria for species demarcation in the genus *Potexvirus* (isolates of different species have less than 72% nucleotide (nt) identity, or 80% amino acid (aa) identity, between their CP or Rep genes) (https://ictv.global/report/chapter/alphaflexiviridae/alphaflexiviridae/potexvirus), revealed ~47% and ~37% aa identity to the Rep and CP of NC_025252, respectively, supporting a wrong taxonomic assignment of the above-mentioned sequences from China available in GenBank.

Given the limited knowledge on the genetic diversity of YVX, a virome investigation was conducted in yam material collected in the state of Bahia (Brazil), using high-throughput sequencing (HTS) on Illumina platform, with the aim to investigate YVX occurrence in *Dioscorea* from South America, and study its diversity.

## Main text

### Methods

#### Collection of yam samples

Leaf samples from symptomatic and asymptomatic *Dioscorea* spp. were collected in commercial cultivation areas in rural zones of Cruz das Almas (n=9) and São Felipe (n=5), as well as from rural home gardens of family-owned properties in Valença (n=9) (Bahia, Brazil) (Additional File 1: Table S1). All sampling activities were conducted with appropriate permission from the landowners. Species identification was confirmed based on morphological characteristics. A total of 23 leaf samples were collected and transported in styrofoam boxes containing ice gel packs to the Virology Laboratory of Embrapa Mandioca e Fruticultura, where they were further processed. Leaf fragments were desiccated in Petri dishes containing CaCl₂, using filter paper as a barrier between the plant material and the desiccant agent for 10 days. After this period, the samples were transferred to Falcon tubes and sent to the Leibniz Institute DSMZ (Braunschweig, Germany) for further processing.

#### HTS and bioinformatics

Total RNA was extracted from desiccated leaf tissue (one RNA extract per sample) using the RNeasy Plant Mini Kit (Qiagen, Germany), according to the manufacturer’s instructions. RNA quality and concentration were assessed by a NanoDrop spectrophotometer (Thermo Fisher Scientific, USA) and electrophoresis on a 1% agarose gel. For initial virome investigations via HTS, the 23 RNA extracts were combined into four pools, considering the geographic region of collection, and taking care of combining equal amount of RNA from each sample (Additional File 1: Table S1). HTS libraries were constructed using the NEBNext UltraExpress® RNA Library Prep Kit (New England Biolabs, USA), and sequenced on an Illumina NextSeq 2000 platform at Leibniz Institute DSMZ. The HTS libraries constructed later in the study from individual yam samples were prepared and sequenced using the same kit and platform. The raw datasets were processed in Geneious Prime v. 2026.0.2 (Dotmatics, USA), according to the in house VirSeq pipeline [16, 17]. In brief, sequencing reads were quality filtered using the following operations and parameters: Trim adapters—kmer length: 27; Trim low quality—minimum quality: 20; Trim adapters based on paired reads overhangs—minimum overlap: 20; Discard short reads—minimum length: 50. After normalization and merging of paired reads, host-related sequence reads were removed by alignment against a *Dioscorea* spp. sequence database (GenBank accessions: chloroplast accessions NC_024170, NC_039707, NC_039836, NC_039851, NC_052854; chromosomes and predicted transcripts Bioproject accession PRJNA695139), using the Geneious mapper at medium–low sensitivity (20% maximum mismatches per read). Sequence reads that did not align to *Discorea* spp. sequences were *de novo* assembled using the Geneious assembler at low sensitivity (10% maximum mismatches per read). For identification and taxonomic assignment, the obtained contigs were aligned locally against custom viral nucleotide and protein sequence databases (generated in September 2023), and verified via online BLAST in the NCBI website.

#### RT-PCR and Sanger sequencing

Reverse Transcription-Polymerase Chain Reactions (RT-PCRs) were performed using a two-step protocol. The first step consisted of complementary DNA (cDNA) synthesis using the HighScriber Reverse Transcriptase Mix (highQu™, Germany). Reactions were prepared in a final volume of 20 µL, including: 4 µL of ALLin™ HighScriber Buffer 5X, 1 µL of primer YVX_1907rev (sequence: 5´-GCGTGAGTTCTTCAGGTCAGA-3´; 10 µM stock), 1 µL of total RNA extract, and 13 µL of distilled water. Subsequently, the samples were subjected to a heating step at 65 °C for 5 min, briefly centrifuged, kept on ice for 1 min, and incubated for 10 min at room temperature. Following addition of 1 µL of the HighScriber Reverse Transcriptase Mix, the samples were incubated at 50 °C for 30 min, followed by enzyme inactivation at 85 °C for 10 min. In the second step, the PCR mix was prepared in a final volume of 50 µL, including: 25 µL of ALLin™ Mega HS HiFi Mastermix, 2X (highQu™), 2 µL of the cDNA template, 2 µL of the forward primer (YVX_1416for [sequence: GCTGAGCCCAAACCGCATGT]), 2 µL of the reverse primer (YVX_1907rev), and 19 µL of PCR-grade water. Each primer was designed on the Rep ORF, and the working solution had a molarity of 10 µM. Amplification was performed according to the following protocol: initial denaturation (95 °C for 1 min); 30 amplification cycles with denaturation at 95 °C for 15 s; annealing at 60 °C for 15 s; extension at 72 °C for 30 s; final incubation at 10 °C. Following gel electrophoresis, the amplification products (expected size: 492 bp) were purified using the Monarch Spin PCR & DNA Cleanup Kit (New England Biolabs) and sequenced by Microsynth (Göttingen).

#### Sequence comparison and phylogenetic analysis

Sequence pairwise alignments were performed in Geneious Prime according to the Clustal Omega algorithm. For the phylogenetic analysis, the CP and Rep aa sequences of the YVX isolates from this study, the NC reference sequence and all other members of the genus *Potexvirus* according to the ICTV Master Species List VMR_MSL41.v1.20260320 were aligned using Clustal Omega (https://www.ebi.ac.uk/jdispatcher/msa/clustalo; accessed on 27.05.2026) [18]. Phylogenetic trees were generated in MEGA12 [19] using the best substitution model and the Maximum Likelihood method. Bootstrap support values were obtained from 1000 replicates.

## Results and discussion

At the beginning of the study in 2025, 23 yam samples were collected across different regions in the state of Bahia. To optimize an initial plant virome exploration, four bulked samples were investigated (Additional File 1: Table S1) by sequencing on a NextSeq 2000 platform, resulting in four datasets composed of ~4 to ~23 M raw reads (Additional File 1: Table S2). Bioinformatic analysis revealed occurrence of contigs assigned to YMV, YMMV, DMaV, badnaviruses and, limited to Pool4, to YVX, which is the subject of this study (Additional File 2: Table S2). The sequence information generated with this first analysis was used for designing a primer pair for screening the individual samples composing the pool, and subsequent RT-PCR reactions revealed amplification of the target YVX sequence only in *D. trifida* (Additional File 1: Table S1). Three infected plants were thereafter selected for a further investigation by HTS on Illumina platform, by sequencing libraries prepared from individual samples (library name: 10B_yam15, 10C_yam21 and 10D_yam22), to generate 15,392,932, 10,884,808 and 12,451,434 total raw reads, respectively (Table 1).

**Table 1:** Generated datasets and results from the bioinformatic analysis on the individual *Dioscorea trifida* samples

Library name	Isolate	Sample origin	Sample preparation	Total raw reads	Trimmed reads	Virus	Completeness	GenBank accession of curated sequence	Reads mapped to curated sequence	Mean coverage
10B_yam15	Y15	Valença	individual plant	15,392,932	14,969,222	YVX	complete genome	PZ482731	581,128	12,993.1x
						YMMV—haplotype 1	complete genome	PZ482746	507,447	7465.5x
						YMMV—haplotype 2	complete cds*	PZ482747	574,840	8453.6x
10C_yam21	Y21	Valença	individual plant	10,884,808	10,577,374	YVX	complete genome	PZ482732	1,461,819	31,795.9x
						YMMV—haplotype 1	complete cds*	PZ482748	83,476	1183.6x
						YMMV—haplotype 2	complete cds*	PZ482749	165,316	2371.2x
						YMV	complete cds*	PZ482750	338,115	4761.0x
10D_yam22	Y22	Valença	individual plant	12,451,434	12,041,826	YVX	complete genome	PZ482733	796,550	17,603.4x
						YMMV—haplotype 1	complete cds*	PZ482751	278,432	4051.9x
						YMMV—haplotype 2	complete cds*	PZ482752	117,546	1688.5x
						YMV	complete cds*	PZ482753	129,019	1863.0x

*cds: coding sequence

The bioinformatic analysis resulted in the assembly of the whole genome sequence of three YVX isolates, named as Y15, Y21 and Y22, of 6141 (Y15 and Y22) and 6142 nt in length, excluding the poly(A) tail, with an average G+C content of 44.9%, 45.4% and 45.4%, respectively. The genome sequences have been annotated and deposited in GenBank under accession number PZ482731, PZ482732, and PZ482733, respectively.

The genome organization resembled the one typical of members of the genus *Potexvirus*, with ORFs annotated as follows: ORF1 ranges from nt position 78 to 4232; ORF2 from nt 4263 to nt 4928; ORF3 from nt 4919 to nt 5245 nt; ORF4 from nt 5172 to nt 5375; ORF5 from nt 5377 to nt 6024. The 5′ and 3′ untranslated regions (UTRs) were determined to be 77 and 117 (Y15, Y22)/118 (Y21) nt in length, respectively.

Sequence comparison at whole-genome level showed 99.7% nt identity between isolates Y21 and Y22, both displaying ~94% nt identity to isolate Y15. Compared to the nuclear core (NC) GenBank reference sequence (NC_025252), originating from Guadeloupe, all three sequences showed ~84% nt identity at whole-genome level (Table 2).

**Table 2:** Percentage identity values at nucleotide (nt) and amino acid (aa) level observed in pairwise comparisons (Clustal Omega implemented in Geneious Prime) of the YVX sequences from this study, and against YVX isolate T551 from Guadeloupe

Genome sequence region	Y15 vs. T551	Y21 vs. T551	Y22 vs. T551	Y15 vs. Y21	Y15 vs. Y22
nt level					
Whole genome	83.9	84.0	83.9	94.6	94.7
Replicase	83.8	84.0	83.9	94.4	94.4
Triple gene block 1	80.6	80.5	80.6	94.0	93.8
Triple gene block 2	85.0	85.6	85.6	95.7	96.3
Triple gene block 3	88.2	86.8	87.3	97.5	98.0
Nucleocapsid protein	84.7	84.1	84.0	94.6	94.6
aa level					
Replicase	94.0	94.4	94.4	98.8	98.8
Triple gene block 1	87.8	87.3	87.3	96.4	96.4
Triple gene block 2	95.4	94.4	95.4	97.2	98.1
Triple gene block 3	92.5	92.5	92.5	100	100
Nucleocapsid protein	94.4	94.9	94.9	99.5	99.5

Comparison of the predicted ORFs of the three newly described sequences to the NC sequence, showed lowest (80.5–80.6% range) and highest (86.8–88.2%) nt identity in the triple gene block (TGB) 1 and TGB3 region, respectively. The CP and Rep region showed % identity in the range 84.0–84.7 and 83.8–84.0, respectively. Among the three YVX sequences from this study, identity values ranged between 93.8% and 98.0% for the different viral genes (Table 2). At aa level, alignment to the NC sequence products, showed identity values in the range 87.3–95.4% for the different viral products, with lowest identity for the TGB1 viral protein. The CP and Rep regions showed % identity in the range 94.4–94.9 and 94.0–94.4, respectively. Pairwise comparison of the three Brazilian isolates showed identities above 99.5%, with 100% identity in the CP aa sequence. The characteristic conserved RNA-dependent RNA polymerase core motif S/TGx3Tx3NS/Tx22GDD [20] was identified in aa position 1207–1242 (TGx3Tx3NTx22GDD) of the viral replicase of the three YVX isolates described in this study, and was identical to the motif in the replicase of the isolate T551 from Guadeloupe.

**Fig. 1:**
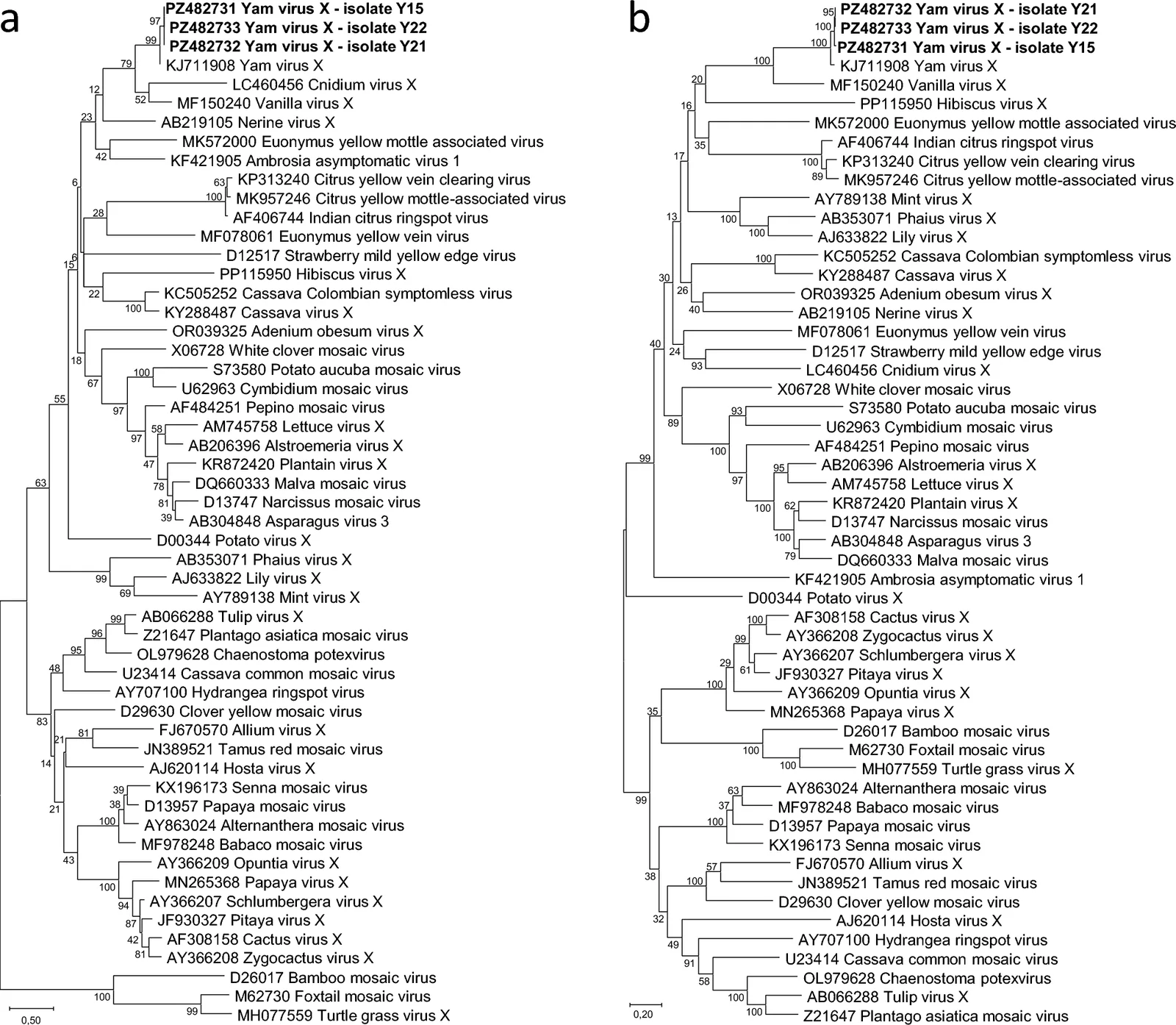
Phylogenetic tree generated in MEGA12 software by the Maximum Likelihood method with 1000 bootstrap replicates and the best substitution model (LG+G+I+F) based on the amino acid sequences of the coat protein (**a**) and replicase (**b**) of the three YVX isolates from this study, the YVX sequence from Guadeloupe (accession KJ815096), and representative members of the genus *Potexvirus* according to ICTV Master Species List VMR_MSL41.v1.20260320. The evolutionary distances are in the units of the number of amino acid substitutions per site. The GenBank accession numbers of the sequences are shown close to the common virus name

A phylogenetic analysis on the CP sequence, including other members of the genus *Potexvirus,* showed clustering of YVX with Vanilla virus X (VVX, *Potexvirus ecsvanillae*) (Fig. 1). The CP and Rep aa sequence showed ~62% and ~60% identity, respectively, by Clustal Omega pairwise alignment against the VVX GenBank accession MF150240.

Inspection of the original pictures from the sampled plants revealed mild leaf chlorosis and, in some leaves, mild mosaic, however symptoms were sometime not apparent, excluding a unique association with YVX presence (Additional File 3: Figure S1). In this context, it is important to remark that abiotic factors may induce symptoms or influence symptom expression, and that other viruses were detected in the individual samples analyzed in this study. In the frame of the virome investigation, the genome sequences of YMV and YMMV isolates were reconstructed and made available in GenBank (Table 1). Although their characterization was not conducted in detail in the present study, the finding is relevant, because mixed viral infections are common in *Dioscorea*. Coinfections involving potyviruses, badnaviruses and other yam-infecting viruses have been reported in yam germplasm and field samples, and such complex viral scenarios may influence symptom expression and complicate its interpretation [21–23]. Biological indexing with YVX in single infection will be needed to determine the symptomatology in *D. trifida* and the host range across other *Dioscorea* species.

Overall, our finding is consistent with the previous report of YVX infecting *D. trifida* in Guadeloupe [15] and expands the current knowledge on its geographic distribution. The detection of YVX only in *D. trifida* in this survey may reflect a biological association with this species, local conditions, or may result from the limited number of samples considered in the study, leading to potential sampling bias. Broader surveys including additional *Dioscorea* species and collection of samples in several yam-producing regions will be required to clarify the host range and incidence of YVX in Brazil. Our results improve the available sequence information on YVX and are expected to enhance detection assays and provide the basis for future studies on the evolution of this virus.

## Limitations

1. Availability of additional sequences will allow broader studies on YVX genetic diversity and support evolutionary studies on introduction pathways and phylogeographic lineages.
2. Given the occurrence of coinfections with other viruses, the association of YVX infection alone to symptom development can not be determined in the context of the study.

## Supplementary Information

Additional file 1: Table S1 Origin of the yam samples collected for the study, composition of the pools considered for the high-throughput sequencing (HTS) investigations, and results of the RT-PCR assays for detection of yam virus X.

Additional file 2: Table S2 Overview of the datasets generated by Illumina sequencing and of the bioinformatic analyses of the four RNA pools investigated at the beginning of the study.

Additional file 3: Figure S1 *Dioscorea trifida* plants sampled during the survey conducted in Valença in 2025, and revealed to be infected with yam virus X by RT-PCR assay. a) Y15; b) Y16; c) Y17; d) Y20; e) Y21; f) Y22; g) Y23. Inspection of the pictures from the sampled plants revealed mild leaf chlorosis and, in some leaves, mild mosaic, however symptoms were sometime not apparent.

## Data Availability

The HTS datasets generated in this study are available from the corresponding author at Leibniz Institute DSMZ under reasonable request. The yam virus X sequences generated in this study have been deposited in GenBank under accession number PZ482731, PZ482732, and PZ482733. The yam mosaic virus sequences generated in this study have been deposited in GenBank under accession number PZ482750, PZ482753. The yam mild mosaic virus sequences generated in this study have been deposited in GenBank under accession number PZ482746, PZ482747, PZ482748, PZ482749, PZ482751, PZ482752. The accession numbers of the virus sequences used in the phylogenetic analysis are reported close to the corresponding branch in the phylogenetic tree (Fig. 1).

## Abbreviations

ORF: Open reading frame
RT: Reverse transcription
PCR: Polymerase chain reaction
UTR: Untranslated region
